# Oral exposure to LaNiO_3_ regulates the immune system, modulates gut flora, and induces intestinal autophagy in mice†

**DOI:** 10.1039/d5na00089k

**Published:** 2025-06-23

**Authors:** Xiaoying Lin, Yanfei Zhang, Qingxuan Liu, Di Wu, Lili Zuo, Yuanbao Zhang, Nianqiu Shi, Rui Chen

**Affiliations:** a Jilin Medical University Jilin 132013 Jilin China linxytime@163.com Shinianqiu2009@163.com; b Beijing Key Laboratory of Occupational Safety and Health, Institute of Urban Safety and Environmental Science, Beijing Academy of Science and Technology Beijing 100054 China chenrui@iuse.ac.cn

## Abstract

LaNiO_3_ exhibits outstanding physical and chemical properties, demonstrating promising potential for regulating immune responses in disease contexts. We discovered that LaNiO_3_ promotes autophagy and immune suppression. After oral administration of LaNiO_3_ in mice (7 days post single exposure at a dose of 10 mg kg^−1^), techniques from metallomics, microbiology, metabolomics, and molecular biology are used to evaluate toxicity, elemental distribution, intestinal autophagy, immune suppression, and effects on intestinal microbiota and metabolites. The results indicate that following a single oral administration of 10 mg per kg LaNiO_3_ to mice over 7 days, both La and Ni primarily accumulated in the gut. LaNiO_3_ suppressed the immune responses through down-regulation of TNF-α and IL-6. Furthermore, LaNiO_3_ increased the abundance of intestinal microbiota and metabolites, with up-regulated microbiota such as *Helicobacter*, *Prevotellaceae*, *Pseudomonas*, *Bacteroides*, *Clostridium sensu stricto 1*, *Ruminiclostridium*, and so on, as well as amino acids and bile acid metabolites such as glutamate, lysine, l-citrulline, and 7α-hydroxy-4-cholesten-3-one. Then, LaNiO_3_ can induce autophagy, including up-regulation of LC3A/B I/II and down-regulation of p62. In summary, oral exposure to LaNiO_3_ in mice regulates the immune system, modulates gut flora, and induces intestinal autophagy. This study provides meaningful data for the safety of LaNiO_3_ oral application and formulation.

## Abstract 2

LaNiO_3_ exhibits outstanding physical and chemical properties, demonstrating promising potential for regulating immune responses in disease contexts.

## Graphical abstract

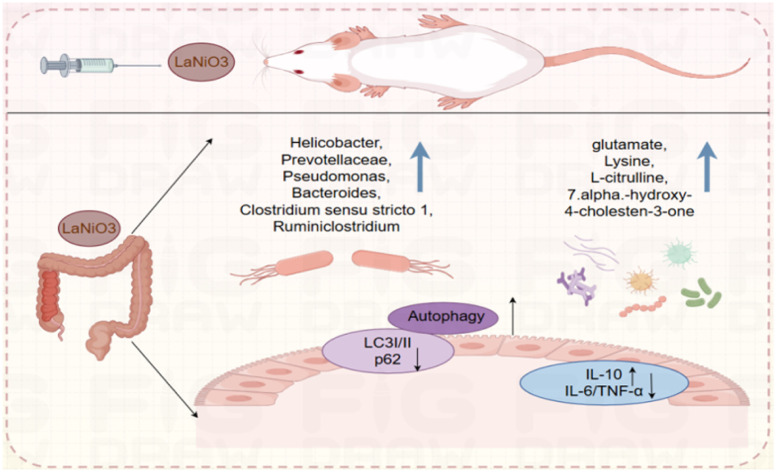

## 1. Introduction

Perovskite nanomaterials (NMs) have an ABX_3_ octahedron crystal structure.^1^ While the A cation is composed of lanthanides including La, Sc, Y, Ce, Pr, Nd, Pm, Sm, *etc.*, the B cation originates from transition elements including Ni, Cr, Co, Fe, *etc.* X is an anion, with halogens and chalcogens being the dominant elements.^2,3^ Perovskite oxide NMs, notably LaNiO_3_, demonstrate excellent physical and chemical properties.^4,5^ Studies indicate that they exhibit peroxidase-like activity, along with antibacterial activity and photocatalytic applications.^6–8^ They can be used as drug delivery carriers. We found that LaNiO_3_ can induce immunological suppression and macrophage autophagy in macrophages.^9^ There have been no reports of its oral application and there is currently no research on its relationship with intestinal delivery or interaction with the intestinal barrier.

Many intestinal metabolites have recently been used as therapeutic drugs, and the gut microbiota can also be used as treatment targets or as early diagnosis indicators.^10,11^ For example, metabolites related to the brain-gut axis can be used to differentiate between the toxicity of methylmercury (MeHg) and inorganic mercury (IHg),^12–14^ as well as the toxicity of nano-selenium and inorganic selenium oral exposure to rats.^15^ Nano-selenium (Se^0^NPs) and fecal transplantation (FMT) can also alleviate the toxicity of MeHg poisoning in rats by regulating or reshaping the gut microbiota.^16,17^ In mice, exposure to nanoplastic polyethylene terephthalate (PET) can cause intestinal obstruction, inducing acute toxicity *via* alterations in lipid metabolism-related metabolites.^18^ Therefore, understanding the regulatory relationship between drugs/nanomedicines/toxic substances and the gut microbiota is critical for clarifying their applications in oral therapy and safety and toxicity mechanisms.

In this research, we use mice as a model; this study assesses toxicity, elemental distribution, intestinal autophagy, immune suppression, and the effects on intestinal microbiota and metabolites seven days after oral administration of LaNiO_3_ (10 mg kg^−1^, single exposure), utilizing techniques from metallomics, microbiology, metabolomics, and molecular biology. This study improves our understanding of the role of LaNiO_3_ in the intestinal barrier of mice, provides a theoretical basis for intestinal barrier targeting applications, and provides more data basis for the biosafety of oral administration.

## 2. Materials and methods

### 2.1 Synthesis and characterization of nanomaterials

LaNiO_3_ nanomaterials were donated by Prof. Hui Wei at Nanjing University and the synthesis and characterization are based on a prior publication.^9^ Briefly, the synthesized LaNiO_3_ was subjected to high-temperature sterilization at 100 °C for 60 minutes. The endotoxin test results showed no endotoxin contamination. Previous studies have found that, according to dynamic light scattering (DLS) measurements, the hydrodynamic size of LaNiO_3_ in water is 350 ± 20 nm. After being exposed to water with a pH of 4.5 and artificial lysosomal fluid (ALF) for 24 hours, the structure of LaNiO_3_ in acidic lysosomes was damaged, presenting a tentacle-like shape. However, such a change did not occur in the water environment. The X-ray diffraction (XRD) pattern confirmed the absence of the characteristic peak 220 in ALF for LaNiO_3_.^9^

### 2.2 Animal experimental design

4 week-old Kunming (KM) mice were divided into 2 groups: the control group and the LaNiO_3_ exposure group, and each group had 5 mice. After adaptation for one week, the mice were fed in metabolic cages; a single oral gavage was adopted, and the LaNiO_3_ (10 mg kg^−1^) was administered to the mice in the gavage group and the same volume of normal saline was gavaged by the control group mice. In this study, we employed two dosage levels; the lower dose was set at 10 mg kg^−1^. It was based on other oral exposure studies, which indicated that the therapeutic dose of nanomaterials *via* oral administration is generally slightly higher than that *via* the blood-based route (5 mg kg^−1^).^19^ As for the higher dose, we set it at 50 mg kg^−1^. This choice was made with reference to the upper limit of therapeutic concentration of nanomaterials in oral administration reported in previous studies.^15^ The living conditions of mice were observed and the survival of mice was recorded. Considering its sustained effect on the regulation of intestinal microbiota metabolites and even on the regulation of intestinal mucosa, we collected fresh feces once (by scratching the back of the mouse to cause stress excretion) on the 7th day after gavage and sealed and stored it at −80 °C. Then, the mice were anesthetized using ether and sacrificed with a broken cone; organs (intestines, stomachs, and other organs) were collected, and the same part of each group of organs was fixed in tissue fixative solution (4% formalin solution) and stored at 4 °C. Following the separation and processing of the intestinal tracts (small intestine: duodenum, jejunum and ileum; large intestine: cecum, colon and rectum) and the contents of the tissue samples from each group, we allocated half of the organ weight from each group for freeze-drying. The remaining half was directly stored in its prototype state at −20 °C for later use.

All applicable agency and/or national animal care and use guidelines were reviewed and approved by the institutional Animal Ethics Committee of Jinlin Medical University (NO. JJKH20210496KJ).

### 2.3 H&E staining

The tissues of mice that were sacrificed on the 7th day or died during breeding were collected and stored at −20 °C. Livers, kidneys, brains, small intestines and cysts were fixed with 4% formalin and embedded in paraffin blocks. Then, the tissues were cut into 4 μm-thick sections using a freezing slicer (Leica CM1860) and mounted onto glass slides. After hematoxylin and eosin (H&E) staining (G1005, Wuhan Google Biotechnology), the pathological changes in tissues were observed and evaluated by a qualified veterinary pathologist under an optical microscope (Leica DM4000M, Germany).

### 2.4 Concentration analysis of La and Ni

samples of the small intestine, large intestine, intestinal contents, brain, kidney, liver and feces were freeze-dried prior to digestion for La and Ni elemental analysis. About 0.1 g freeze-dried samples were digested using concentrated nitric acid overnight. The solutions were then heated at 120 °C to remove the remaining nitric acid. The remaining solutions were diluted to 4 mL with 2% (v/v) HNO_3_ containing 0.1% (v/v) β-mercaptoethanol. The concentrations of La and Ni were analyzed by Thermo X7 inductively coupled plasma-mass spectrometry (ICP-MS).^9–11^ Fish muscle (ORT-2, National Research Council of Canada) was used for quality control with a recovery rate of 90–110%.

### 2.5 Western blotting

The gut in mice of control and LaNiO_3_ groups after exposure for 7 days was determined using a BCA protein assay kit (Beyotime, China). The protein expression of p62 and LCA/B in guts was tested. The concentration of each sample was determined using a BCA protein assay kit (Beyotime, China). After centrifuging for 20 min at 13 500 rpm, equal amounts (35 μg) of proteins were separated by electrophoresis on 10% sodium dodecyl sulphate polyacrylamide (SDS-PAGE) gels, and these separated protein bands were transferred to 0.22 μM PVDF membranes. The membranes were blocked with 5% (w/v) skimmed milk powder in Tris-buffered saline containing 0.1% (v/v) Tween-20 (TBST) for 2 h at room temperature. Then, all the antibodies were probed with dilution at 1 : 1000 at 4 °C overnight. Primary antibodies included p62 (GB11531, Servicebio), LCA/B (GB11124-100, Servicebio) and GAPDH (60004-1-Ig, Proteintech). After washing with TBST, anti-mouse IgG HRP-linked antibody (BL063A) at the concentration of 1 : 10 000 for 2 h at room temperature. After washing by TBST again, the blots were developed using an ECL Plus kit (Beyotime, China) and visualized using a molecular imager (Amersham QuantStudio 3, Thermo Fisher, USA) with image lab software.

### 2.6 Serum biochemical analysis

Serum biochemical indicators, including alanine aminotransferase (ALT), glutamic oxaloacetic transaminase (AST), uric acid (UREA), creatinine (Crea), glucose (Glu), and cholesterol (Chol), were quantified using a veterinary microfluidic liver and kidney function test kit (RS20110, Nanjing Huaren Biotechnology Co., Ltd.).

### 2.7 Real-time reverse transcription quantitative PCR (real-time RT-qPCR) and inflammation effects

0.1 mg small gut (ileum) was used RT-PCR experiments were done to determine the level of mRNA expression. The TRIzol reagent method (Life Technology, CA, USA) was utilized to isolate RNA from cells. About 10 pmol oligonucleotide (Oligo dT) (Sigma, USA) primer and Moloney murine leukemia virus reverse transcriptase (M-MLV, Promega, Madison, USA) were added to 2 mg of RNA to generate cDNA. Each sample was prepared for real-time quantitative PCR in a final reaction volume of 20 mL by adding Master Mix (Promega, Madison, USA) and SYBR Green (Invitrogen, Paisley, UK). The amplification cycle was performed using a Realplex4 (Eppendorf, Germany). The primers in this study were synthesized by Sangon Biotech: TNFα: F-CATCTTCTCAAAATTCGAGTGACAA, R-TGGGAGTAGACAAGGTACAACCC; IL10: F-CTTACTGACTGGCATGAGGATCA, R-GCAGCTCTAGGAGCATGTGG; IL-6: F-GAGGATACCACTCCCAACAGACC, R-CAAGCAGAACTGAACTACCATCG; GAPDH: F-GACCCCTTCATTGACCTCAAC, R-CTTCTCCATGGTGGTGAAGA.

### 2.8 16S rDNA gene sequencing

High-throughput sequencing of bacterial 16S rDNA genes using fecal samples collected from each group on day 7 was performed as described previously.^12–14^ Briefly, microbial DNA in fecal samples was isolated, quantified and amplified. The samples were barcoded and pooled to construct the sequencing library, and finally, high-throughput sequencing analysis was performed. Metastats software (https://metastats.cbcb.umd.edu) was applied to compare the changes in the gut microbiota among different groups. More details can be found in the ESI.† Metastats software was applied (http://metastats.cbcb.umd.edu/) to compare species richness between groups. Please refer to the ESI† for more details.

### 2.9 Metabolomics profiling

The fresh fecal samples collected from each group on day 7 (1 g) were subjected to liquid chromatography-mass spectrometry (LC-MS) analysis as described previously.^12–14^ Briefly, fresh fecal samples were dissolved in pre-cooled 50% methanol and the separation of the supernatants was performed using an ultra-performance liquid chromatography (UPLC) system (SCIEX, UK). A high-resolution tandem mass spectrometer TripleTOF5600plus (SCIEX, UK) was used to detect metabolites eluted from the column. The XCMS Online tool was used to pick up and align peaks and calculate the accumulated peak intensity. UPLC software (Waters ACQUITY UPLC HSS T3 C18, USA) was used to quantify the metabolite concentrations. More details can be found in the ESI.†

### 2.10 Bioinformatic analysis

For 16S rDNA analysis, STAMP software was utilized to confirm differences in the abundances of individual taxonomy between the two groups. The LDA effect size (LEfSe) was used for the quantitative analysis of biomarkers within different groups. This method was designed to analyze data in which the number of species is much higher than the number of samples and to provide biological class explanations to establish statistical significance, biological consistency, and effect-size estimation of predicted biomarkers. To identify differences in microbial communities between the two groups, ANOSIM and ADONIS were performed based on Bray–Curtis dissimilarity distance matrices.

For metabolomics analysis, the processed data were analyzed using the R package (ropls) after sum normalization, where it was subjected to multivariate data analysis, including Pareto-scaled principal component analysis (PCA) and orthogonal partial least-squares discriminant analysis (OPLS-DA). 7-fold cross-validation and response permutation testing were used to evaluate the robustness of the model. The variable importance in the projection (VIP) value of each variable in the OPLS-DA model was calculated to indicate its contribution to the classification. Student's *t*-test was applied to determine the significance of differences between two groups of independent samples. VIP > 1 and a *p* value <0.05 were used to screen significant changed metabolites. Pearson's correlation analysis was performed to determine the correlation between two variables.

### 2.11 Statistical analysis

Statistical tests were performed using Origin 9.0 software and *p* < 0.05 was considered to be significant. All data were presented as means ± standard deviations (SDs). A *t*-test was used to compare means among groups.

## 3. Results

### 3.1 Organ pathology and elemental distribution

**Fig. 1:**
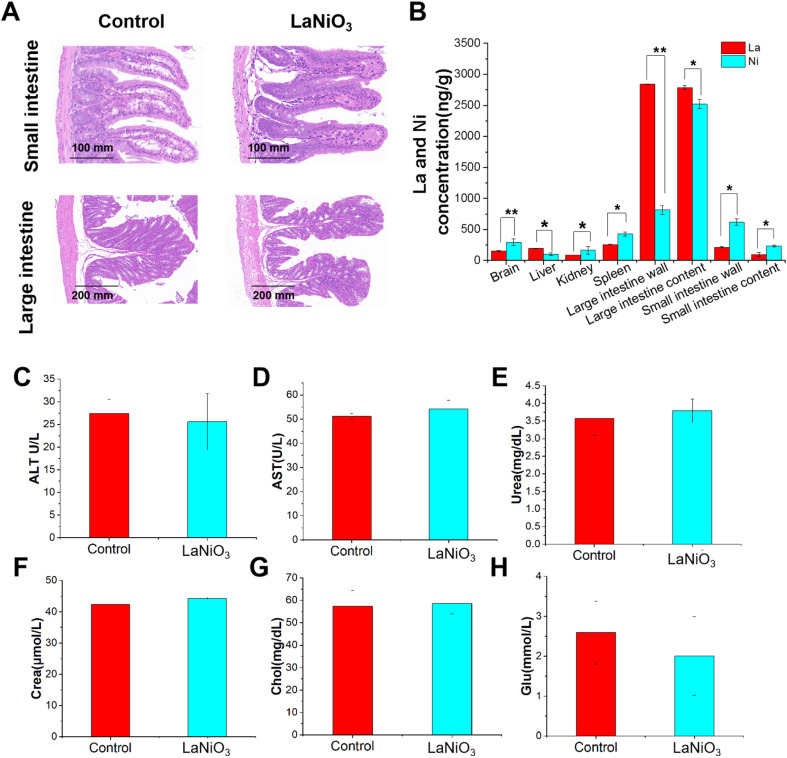
Organ pathology and elemental distribution. (A) H&E staining of the small intestine (ileum) (100×) and large intestine (colon) (50×) in the control and LaNiO_3_ (10 mg kg^−1^) (*n* = 5) groups. (B) The concentrations of La and Ni elements in mice tissue samples after 7 d of single oral exposure to 10 mg per kg LaNiO_3_. *p < 0.05, **p < 0.01: La *vs* Ni. Serum ALT (C), AST (D), urea (E), crea (F), chol (G), and glu (H) were quantified using a veterinary microfluidic liver and kidney function test kit (*n* = 5).

Pathological results showed that when compared with the control group, the LaNiO_3_ exposed mice showed no villus injury or inflammatory infiltration in the intestine (Fig. 1A), as well as no damage in other organs (Fig. S1†). Furthermore, body weight was not significantly different from the control group (Fig. S2†). These results indicate that LaNiO_3_ has good biocompatibility after oral administration at a dose of 10 mg kg^−1^.

ICP-MS was used to analyze the distribution and accumulation of La and Ni elements in mouse organs after 7 d of single oral exposure to LaNiO_3_ (10 mg kg^−1^). The results show that most of the La and Ni accumulated in the gut, including the large/small intestine wall and mainly in the contents (Fig. 1B) (*p* < 0.01, *p* < 0.001), and only small amounts of La and Ni accumulated in the organs, such as the brain, liver, kidneys and spleen. This suggests that the gut might be the primary target organ for LaNiO_3_. In addition, compared with La element in the organs, there is a higher Ni concentration in the organs (*p* < 0.05, *p* < 0.01).

The serum ALT level (C), AST level (D), urea level (E), crea level (F), chol level (G), and glu level (H) were detected using reagent kits. All results show no significant difference compared to the control group, indicating that LaNiO_3_ has good biocompatibility.

### 3.2 LaNiO_3_ promotes gut immune suppression

**Fig. 2:**
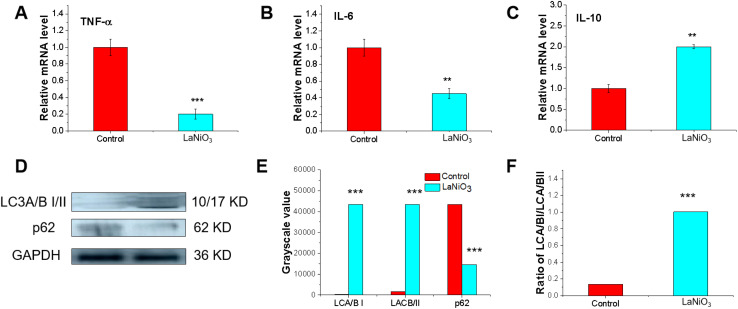
LaNiO_3_ regulates immunological responses in mice small gut tissue. The mRNA levels of IL-10 (A), TNF-α (B), and IL-6 (C) in the gut (ileum) after 7 days of single oral exposure to 10 mg per kg LaNiO_3_ (*n* = 5) **p < 0.01, ***p < 0.001: LaNiO_3_*vs* control. (D) Western blot of LC3A/B and p62 in the small intestine (ileum); (E) protein grayscale value reading using ImageJ software. ***p < 0.001: LaNiO_3_*vs* control; (F) grayscale ratio LC3A/BI/LC3A/BII determined using ImageJ software. ***p < 0.001: LaNiO_3_*vs* control.

The results of genes express texted by RT-PCR were shown that oral exposure to LaNiO_3_ in mice, the pro-inflammatory cytokines, such as TNF-α (Fig. 2A), IL-6 (Fig. 2B) are decreased at the mRNA level (**p < 0.01, ***p < 0.001), while promotes the expression of the anti-inflammatory cytokine IL-10 in the gut (**p < 0.01) (Fig. 2C). These results indicate that LaNiO_3_ has immunosuppressive effects.

Fig. 2D and E show the expression of the autophagy-related proteins LC3A/B and p62. In comparison to the control group, LC3A/B in the gut of the LaNiO_3_ exposure group was up-regulated (***p < 0.001), while the p62 protein was down-regulated (***p < 0.001). The ratio of LC3A/BI/LCA/BII in the LaNiO_3_ exposure group was also increased (***p < 0.001) (Fig. 2F). This means that LaNiO_3_ induces gut autophagy in mice following oral exposure at a dose of 10 mg kg^−1^.

### 3.3 Intestinal microbes

**Fig. 3:**
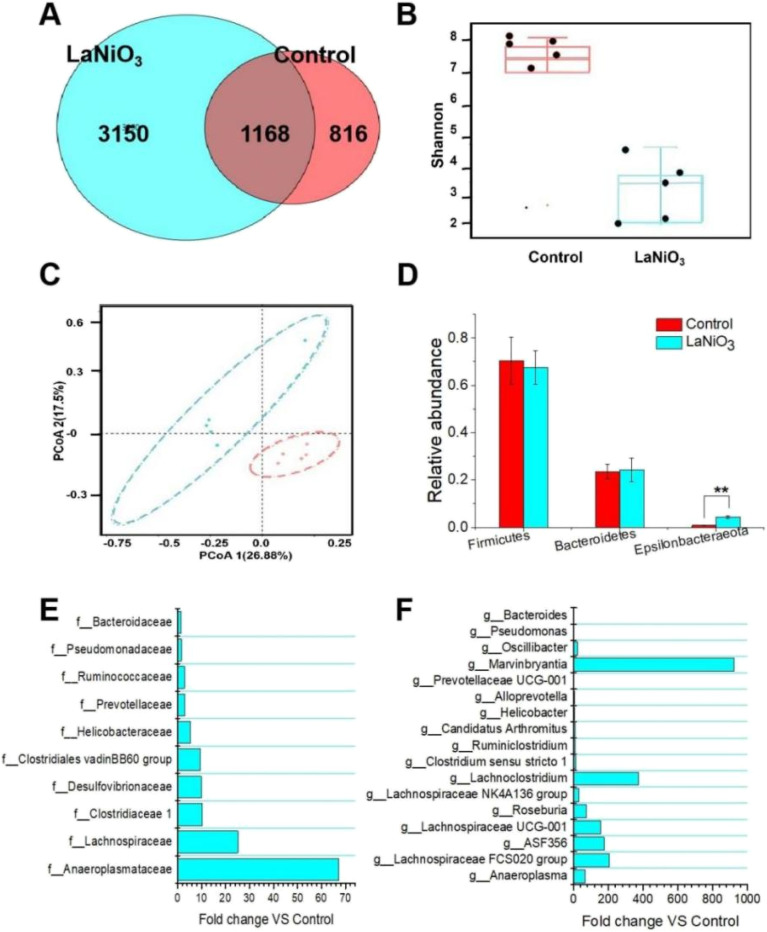
Impacts on the gut microbiota in mice 7 d after single oral exposure to 10 mg per kg LaNiO_3_. (A) OTU changes; (B) α diversity analysis; (C) β diversity analysis PCA analysis; (D) the phylum level changes (relative abundances >0.05%). **p < 0.01: LaNiO_3_*vs* the control group. (E) The family level changes (fold changes >2, LaNiO_3_*vs* the control group). (F) The genus level changes (fold changes >2, LaNiO_3_*vs* the control group) (*n* = 5).

Fig. 3 shows the changes in the gut microbiota in feces on day 7 after oral exposure to LaNiO_3_. The operational taxonomic unit (OTU) was used to classify the gut microbiota, as shown in Fig. 3A. Compared to the control group, the LaNiO_3_ group had 3150 different OTUs, indicating that LaNiO_3_ changed the OTUs of normal species. The α diversity index can reflect species richness. Fig. 3B shows the Shannon α diversity index, with a box chart illustrating the median, dispersion, maximum, minimum and outliers of species diversity across groups. The difference index between the LaNiO_3_ group and the control group indicates that their diversity was different.

The β diversity index can reflect habitat diversity; the β diversity index PcoA is presented in Fig. 3C. There were some similarities and differences in the three groups, with differences in the dimensions of PcoA1 (26.88%) and PcoA2 (17.5%), indicating that the groups showed normal habitat diversity. Fig. 3D shows the gut microbiota composition at the phylum level. Compared to the control group, LaNiO_3_ did not significantly affect *Firmicutes* and *Bacteroidetes* (*p* > 0.05), while it significantly increased *Epsilonbacteraeota* (*p* < 0.01).

Fig. 3E shows the abundance of the gut microbiota at the family level (*vs* control >1.5). LaNiO_3_ increased the abundance of various microbial classes, including *Anaeroplasmataceae* (67.2), *Lachnospiraceae* (25.12), *Clostridiaceae 1* (10.23), *Desulfovibrionaceae* (9.89), *Clostridiales vadin* BB60 group (9.4), *Helicobacteraceae* (5.4), *Prevotellaceae* (2.97), *Ruminococcaceae* (2.89), *Pseudomonadaceae* (1.62), and *Bacteroidaceae* (1.48). See Annex 1 for more details on changes in the gut microbiota at the family level.

Fig. 3F demonstrates the abundance of the gut microbiota at the genera level (*vs* control >1.5). LaNiO_3_ increased the abundance of various microbial classes, including *Anaeroplasma* (67.34), *Lachnospiraceae* FCS020 group (206.64), ASF356 (176.2), *Lachnospiraceae* UCG-001 (155.34), *Roseburia* (73.65), *Lachnospiraceae* NK4A136 group (30.37), *Lachnoclostridium* (372.98), *Clostridium sensu stricto 1* (12.43), *Ruminiclostridium* (9.69), *Candidatus* Arthromitus (9.32), *Helicobacter* (5.44), *Alloprevotella* (4.46), *Prevotellaceae* UCG-001 (1.54), *Marvinbryantia* (924.65), *Oscillibacter* (24.8), *Pseudomonas* (1.62), and *Bacteroides* (1.47). See Annex 1 for more details on changes in the gut microbiota at the genera level.

### 3.4 Metabolites

**Fig. 4:**
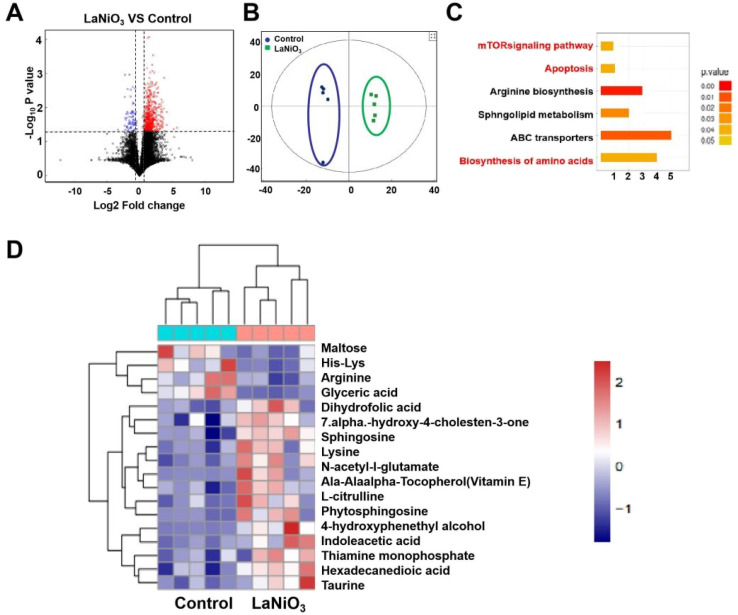
Changes in the profile of metabolites in mice feces 7 days after single dose oral exposure to10 mg per kg LaNiO_3_. (A) Univariate statistical analysis of metabolites in fecal samples in the LaNiO_3_*vs* the control group (fold >2 and *p* < 0.05). (B) PCA analysis. (C) The KEGG pathway changes in feces. (D) Cluster analysis of differential metabolites using stratified cluster heat maps constructed with 2-fold variation of molecular characteristics (*p* < 0.05) (*n* = 5).

The LC-MS technique was used to investigate metabolite profile changes in the feces of LaNiO_3_-exposed mice. Fig. 4 illustrates the metabolome changes (volcano map, PCA analysis, cluster analysis, and specific differential metabolites) in fresh feces from the LaNiO_3_ group and the control group.

In Fig. 4A, exposure to LaNiO_3_ resulted in 769 metabolites, of which 58 metabolites (47 increased and 11 decreased, >1.5 fold, and *p* < 0.05) were significantly different from the control group. These findings suggested that the metabolic disorder was caused by LaNiO_3_ exposure.

The PCA results are shown in Fig. 4B. The LaNiO_3_ group is closer in spatial position to the control groups, indicating that LaNiO_3_ has an impact on metabolic abundance of feces.

Fig. 4C shows the results of the KEGG pathway. LaNiO_3_ changes feces metabolism, including the mTOR signaling pathway, apoptosis and biosynthesis of amino acids.

Fig. 4D shows a cluster analysis of differential metabolites using stratified cluster heat maps with 2-fold variance in molecular characteristics (*p* < 0.05). The color blocks of the LaNiO_3_ group were significantly different from the control group. This indicates that LaNiO_3_ has an impact on metabolism. LaNiO_3_ increased the levels of lysine, *N*-acetyl-l-glutamate, and indolelactic acid. More detailed information on metabolite changes is shown in Table S2.†

### 3.5 Correlation between the gut microbiota and metabolites

**Fig. 5:**
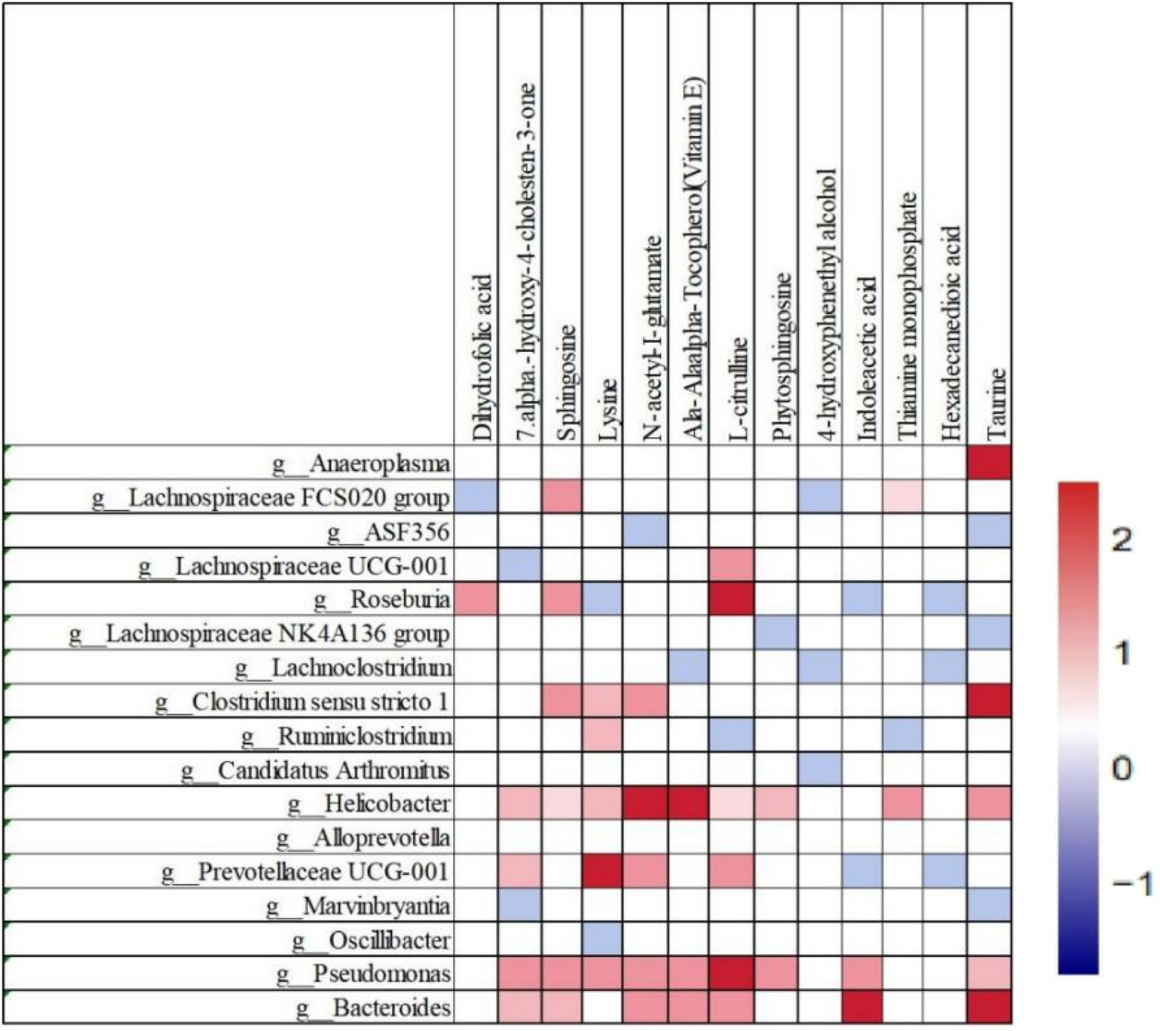
Spearman correlation analysis between the gut microbiota (at genus levels) and metabolites. The blue color represents a negative correlation and the maximum correlation coefficient is −2, whereas the red color represents a positive correlation, and the maximum positive correlation coefficient is 2 (*n* = 3).

Spearman correlation analysis was used to dissect the association between metabolite changes and the gut microbiota in feces on day 7 in the LaNiO_3_ group (Fig. 5). The results show that changes in intestinal metabolites are partially correlated with changes in the aforementioned gut microbes.

Upregulation of 7alpha-hydroxy-4-cholesten-3-one correlates positively with *Helicobacter*, *Prevotellaceae*, *Pseudomonas*, and *Bacteroides*. Indoleacetic acid has a positive correlation with *Pseudomonas* and *Bacteroides*. Lysine exhibits a positive correlation with *Clostridium sensu stricto 1*, *Ruminiclostridium*, *Helicobacter*, and *Pseudomonas*. l-Citrulline has a positive correlation with *Lachnospiraceae*, *Helicobacter*, *Prevotellaceae*, *Pseudomonas*, and *Bacteroides*. *N*-Acetyl-l-glutamate has a positive correlation with *Clostridium sensu stricto 1*, *Helicobacter*, *Prevotellaceae*, *Pseudomonas*, and *Bacteroides*. Taurine has a positive correlation with *Anaeroplasma*, *Clostridium sensu stricto 1*, *Helicobacter*, *Pseudomonas*, and *Bacteroides*.

## 4. Discussion

### 4.1 Mice orally exposed to LaNiO_3_ show high accumulation in the intestine and good biocompatibility

After oral administration, nanomaterials first pass through the gastrointestinal tract, which is the first organ of action. The pathway and degree of absorption through the intestine are determined by the physicochemical properties of the nanomaterials, such as particle size.^20^ LaNiO_3_ has a primary size of around 350 nm, which is a relatively large nanometer size. Due to its large size, it may be mainly retained in the intestine, with a small fraction absorbed into the bloodstream *via* the intestine.

Serum ALT and AST levels reflect the degree of liver cell damage,^21^ while urea and crea indicate renal impairment.^21^ The chol level reflects overall lipid metabolism,^22^ and the glu level correlates with glucose metabolism.^22^ The above indicators overall demonstrate that oral administration of LaNiO_3_ has no impact on the systemic health of mice and exhibits good biocompatibility.

### 4.2 LaNiO_3_ promotes gut immune suppression and induces gut autophagy

TNF-α is mainly secreted by macrophages and plays an important role in the onset of inflammation. It also regulates the tumor microenvironment and the development of diseases.^23^ IL-6 is produced by macrophages and has pleiotropic functions in the immune system, including activating immune cells to remove pathogens, repairing damaged tissues, and regulating acute immunological responses. It is also involved in autoimmune diseases and chronic inflammation.^24^ IL-10, an anti-inflammatory cytokine, is widely involved in various pathological processes, including human tissue destruction, edema formation, and inflammatory responses.^25^ In summary, the decreased expression of proinflammatory cytokines suggests that LaNiO_3_ may serve as a potential inhibitor for inflammation therapy.

Autophagy is a mechanism for the degradation and recycling of damaged organelles and proteins for reuse. It can be activated in various stress states, including hunger, poisoning, and cancer, to maintain the stability of the intracellular environment. Autophagy plays a crucial role in the processes of removal, degradation, and recycling of misfolded proteins and damaged organelles. When cells detect signals of misfolded proteins and damaged organelles, autophagy is triggered and autophagosomes develop. These are a class of vesicles composed of a double-layer membrane with engulfed matter.^26^ In an acidic environment, autophagosomes fuse with lysosomes to form autophagolysosomes, and the contents of autophagolysosomes will be degraded by digestive enzymes in the lysosomes.^27^

Previous results of *in vitro* LaNiO_3_ exposure to macrophages showed that under the stimulation of acidic lysosomes, LaNiO_3_ can induce the release of La and Ni ions from macrophage lysosomes, leading to autophagy and further immunosuppressive effects.^9^ In this study, we found that LaNiO_3_ can accumulate at high concentrations in the intestines of mice, probably in intestinal cells, and enter other organs in modest amounts. Endothelial cells, macrophages, goblet cells, and other mucosal barriers are primarily responsible for the absorption of foreign substances in the intestine.^28^ The overall state of intestinal wall cells reflects their ability to respond to LaNiO_3_. Furthermore, we demonstrated autophagy at the protein level by analyzing the conversion of the autophagy-related protein microtubule-associated protein 1 light chain 3 (LC3). LC3 has two isoforms: LC3-I is cytosolic and LC3-II is associated with autophagosome membranes. Autophagy is characterized by an increase in the LC3-II protein ratio.^29^ WB results show that the level of LC3-II and the ratio of LC3-II to LC3-I expression increased as the dosage of LaNiO_3_ increased. As we all know, p62, also known as SQSTM1/sequestosome 1, is a substrate that is preferentially degraded during autophagy.^30^ As the concentration of LaNiO_3_ increased, the expression of p62 decreased. Taken together, LaNiO_3_ induced autophagy in the intestines of mice.

The accumulation of La and Ni elements in organs also has important significance at the cellular and tissue levels. La element has extremely low levels in the body and is used in certain radiopharmaceutical research, as well as a candidate drug in tumor treatment.^31^ In addition, Ni is an essential trace element for the human body and can participate in catalytic reactions of certain enzymes, such as urease and hydrogenase,^32^ which are involved in biological processes such as energy metabolism and nitrogen metabolism, as well as in some microbial transformation processes.^33^ This study found that the concentration of Ni in the body is higher than that of La and LaNiO_3_ is prone to undergoing chemical structural changes in acidic lysosomal environments. The intestinal environment, particularly the small intestine close to the stomach, is also acidic, with a pH of 4.5. Therefore, it is speculated that the acidic environment of the small intestine may induce chemical structural changes in LaNiO_3_, leading to the release of Ni and La ions to regulate the intestinal immune responses.

### 4.3 LaNiO_3_ modulates the gut microbiota and metabolites

Mice oral exposure to LaNiO_3_ can regulate various gut microbiota and metabolites related to anti-inflammatory, immunosuppressive, and autophagy effects *via* modulating KEGG pathways such as amino acid and bile acid synthesis.

LaNiO_3_ increases amino acid synthesis metabolites including glutamate, lysine, and l-citrulline. Lysine suppresses protein degradation through the autophagic-lysosomal system in C2C12 myotubesis^34^ and has a positive correlation with *Clostridium sensu stricto 1*,^35^*Ruminiclostridium*,^36^*Helicobacter*,^37^ and *Pseudomonas*.^38^l-Citrulline regulates ASS1-mediated metabolic reprogramming and promotes macrophage inflammatory polarization in viral myocarditisis,^39^ and it has a positive correlation with *Lachnospiraceae*,^40^*Helicobacter*,^41^*Prevotellaceae*,^42^*Pseudomonas*^43^ and *Bacteroides*.^44^*N*-Acetyl-l-glutamate inhibits programmed cell death in a chronic MPTP mouse model of Parkinson's diseaseis,^45^ and has a positive correlation with *Clostridium sensu stricto 1*,^46^*Helicobacter*,^47^*Prevotellaceae*,^48^*Pseudomonas*,^49^ and *Bacteroides*.^50^

In addition, LaNiO_3_ can up-regulate bile acid synthesis metabolites including 7alpha-hydroxy-4-cholesten-3-one. Cholesterol induces autophagy *via* the IRE1/JNK pathway by promoting autophagic cell death in heart tissue.^51^ Up-regulation of 7alpha-hydroxy-4-cholesten-3-one correlates positively with *Helicobacter*,^52^*Prevotellaceae*^42^*Pseudomonas*^53^ and *Bacteroides*.^54^

We mainly studied the correlation between the gut microbiota and metabolites and found that LaNiO_3_ can promote an increase in the abundance of metabolites related to intestinal amino acid and bile acid metabolism. These metabolites within the relevant pathways play a positive regulatory role in intestinal autophagy and further regulate intestinal immunity.

## 5. Conclusion

**Fig. 6:**
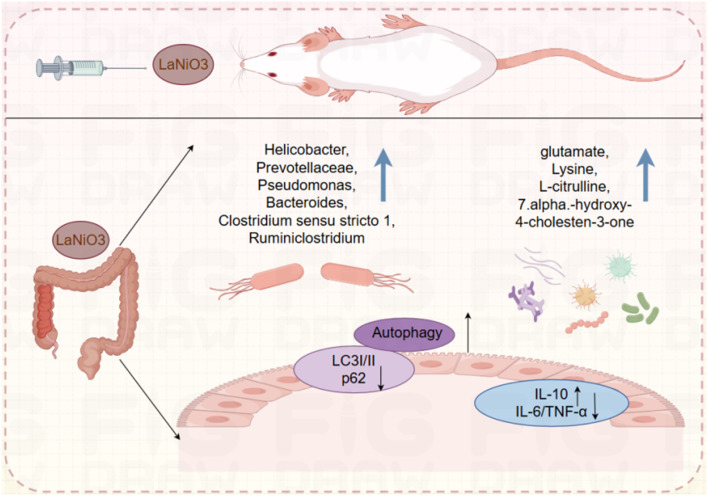
Mechanism diagram of the biological effects after oral exposure to LaNiO_3_ in mice.

The results indicate that following a single oral administration of 10 mg per kg LaNiO_3_ to mice over 7 days, La and Ni mainly accumulated in the gut and LaNiO_3_ can suppress the immune system in the gut by down-regulation of TNF-α and IL-6. Furthermore, LaNiO_3_ increased the abundance of intestinal microbiota and metabolites, with up-regulated microbiota such as *Helicobacter*, *Prevotellaceae*, *Pseudomonas*, *Bacteroides*, *Clostridium sensu stricto 1*, *Ruminiclostridium*, and so on, as well as metabolites such as amino acids and bile acids: glutamate, lysine, l-citrulline, and 7alpha-hydroxy-4-cholesten-3-one. Then, LaNiO_3_ can induce autophagy, including up-regulation of LC3A/B I/II and down-regulation of p62. In summary, oral exposure to LaNiO_3_ in mice suppresses the immune system, modulates gut flora, and induces intestinal autophagy (Fig. 6). This study provides meaningful data for the safety of LaNiO_3_ oral application and formulation.

## Conflicts of interest

The authors declare no competing interests.

## Supplementary Material

NA-OLF-D5NA00089K-s001

## Data Availability

The data supporting this article “Oral exposure to LaNiO_3_ regulates the immune system, modulates gut flora, and induces intestinal autophagy in mice” have been included as part of the ESI.†
