# Supplementary material for: Oral exposure to LaNiO3 regulates the immune system, modulates gut flora, and induces intestinal autophagy in mice
Source: Nanoscale Adv. 2025 Jun 23;7(16):5007–18. doi: 10.1039/d5na00089k (PMC12242274; doi:10.1039/d5na00089k)
Supplement: NA-007-D5NA00089K-s001 [file NA-007-D5NA00089K-s001.pdf]

Supplementary Information (SI) for Nanoscale Advances.
This journal is © The Royal Society of Chemistry 2025

Oral exposure to $LaNiO_3$ regulates the immune system, modulates gut flora, and induces intestinal autophagy in mice

Xiaoying Lin**[1], Yanfei Zhang[1], Qingxuan Liu[1], Di Wu[1], Lili Zuo[1], Yuanbao Zhang[2], Nianqiu Shi[1]*, Rui Chen[2]***

1 Jilin Medical University, Jilin 132013, Jilin, China

2 Beijing Key Laboratory of Occupational Safety and Health, Institute of Urban Safety and Environmental Science, Beijing Academy of Science and Technology, Beijing 100054, China.

*Corresponding authors:

*Xiaoying Lin, E-mail: linxytime@163.com

**Nianqiu Shi, E-mail: Shinianqiu2009@163.com

***Rui Chen, E-mail: chenrui@iuse.ac.cn

1. Materials and Methods

1.1 16SrRNA

1.1.1 Sequencing

（1）**Extraction of genome DNA**

Total genome DNA from samples was extracted using CTAB/SDS method. DNA concentration and purity was monitored on 1% agarosegels. According to the concentration, DNA was diluted to 1ng/μl using sterile water.

（2）**Amplicon Generation**

Primer：16S V3-V4: 341F-806R, 18S V9: 1380F-1510R, ITS1: ITS1F- ITS2R. 16S /18S rRNA genes were amplified used the specific primer with the barcode. All PCR reactions were carried out in 30μL reactions with 15μL of Phusion®High-Fidelity PCR Master Mix (New England Biolabs); 0.2μM of forward and reverse primers, and about 10 ng template DNA.Thermal cycling consisted of initial denaturation at 98 ℃ for 1 min, followed by 30 cycles of denaturation at 98℃ for 10 s, annealing at 50 ℃ for 30 s, and elongation at 72 ℃ for 60 s. Finally 72 ℃ for 5 min.

（3）**PCR Products quantification and qualification**

Mix same volume of 1X loading buffer (contained SYB green) with PCR products and operate electrophoresis on 2% agarose gel for detection. Samples with bright main strip between 400-450bp were chosen for further experiments.

（4）**PCR Products Mixing and Purification**

PCR products was mixed in equidensity ratios. Then, mixture PCR products was purified with AxyPrepDNA Gel Extraction Kit (AXYGEN).

（5）**Library preparation and sequencing**

Sequencing libraries were generated using NEB Next®Ultra™DNA Library Prep Kit for Illumina (NEB, USA) following manufacturer’s recommendations and index

codes were added. The library quality was assessed on the Qubit@ 2.0 Fluorometer (Thermo Scientific) and Agilent Bioanalyzer 2100 system. At last, the library was sequenced on an Illumina Miseq/HiSeq2500 platform and 250bp/300bp paired-end reads were generated.

1.1.2 Data analysis

（1）**Paired-end reads assemblies**

Paired-end reads from the original DNA fragments were merged using FLASH, a very fast and accurate analysis tool, which was designed to merge paired-end reads when at least some of the reads overlap the read generated from the opposite end of the same DNA fragment. Paired-end reads was assigned to each sample according to the unique barcodes.

（2）OTU cluster and Species annotation

Sequences analysis were performed by UPARSE software package using the UPARSE-OTU and UPARSE-OTUref algorithms. In-house Perl scripts were used to analyze alpha (within samples) and beta (among samples) diversity. Sequences with ≥97% similarity were assigned to the same OTUs. We pick a representative sequences for each OTU and use the RDP classifier to annotate taxonomic information for each representative sequence. In order to compute Alpha Diversity, we rarify the OTU table and calculate three metrics: Chao1 estimates the species abundance; Observed Species estimates the amount of unique OTUs found in each sample, and Shannon index. Rarefaction curves were generated based on these three metrics.

（3）**Phylogenics distance and community distribution**

Graphical representation of the relative abundance of bacterial diversity from phylum to species can be visualized using Krona chart. Cluster analysis was preceded by principal component analysis (PCA), which was applied to reduce the dimension

of the original variables using the QIIME software package. QIIME calculates both weighted and unweighted unifrac distance, which are phylogenetic measures of beta diversity. We used unweighted unifrac distance for Principal Coordinate Analysis (PCoA) and Unweighted Pair Group Method with Arithmetic mean (UPGMA) Clustering. PCoA helps to get principal coordinates and visualize them from complex, multidimensional data. It takes a transformation from a distance matrix to a new set of orthogonal axes. By which the maximum variation factor is demonstrated by first principal coordinate, and the second maximum one by the second principal coordinate, and so on. UPGMA Clustering is a type of hierarchical clustering method using average linkage and can be used to interpret the distance matrix.

1.2 Metabolomics profiling

1.2.1 Chemicals

Ammonium acetate (NH4AC) was purchased from Sigma Aldrich, Acetonitrile was purchased from Merck, ammonium hydroxide (NH4OH) and methanol were purchased from Fisher.

1.2.2 Sample Collection and Preparation

The mice feces were quickly frozen in liquid nitrogen immediately after dissection. Then the tissue were cut on dry ice (~80 mg) into an Eppendorf tube (2 mL). The tissue samples with 200 μL of H2O and five ceramic beads were homogenized using the homogenizer. 800 μL methanol/acetonitrile (1:1, v/v) were added to homogenized solution for metabolite extraction. The mixture was centrifuged for 15 min (14000 g, 4 °C). The supernatant was dried in a vacuum centrifuge. For LC-MS analysis, the samples were re-dissolved in 100 μL acetonitrile/water (1:1, v/v) solvent.

1.2.3 LC-MS/MS Analysis

Analysis was performed using an UHPLC (1290 Infinity LC, Agilent Technologies) coupled to a quadrupole time-of-flight (AB Sciex TripleTOF 6600) in Shanghai Applied Protein Technology Co., Ltd. For HILIC separation, samples were analyzed using a 2.1 mm × 100 mm ACQUIY UPLC BEH 1.7 μm column (waters, Ireland). In both ESI positive and negative modes, the mobile phase contained A=25 mM ammonium acetate and 25 mM ammonium hydroxide in water and B= acetonitrile. The gradient was 85% B for 1 min and was linearly reduced to 65% in 11 min, and then was reduced to 40% in 0.1 min and kept for 4 min, and then increased to 85% in 0.1 min, with a 5 min re-equilibration period employed.

For RPLC separation, a 2.1 mm × 100 mm ACQUIY UPLC HSS T3 1.8 μm column (Waters, Ireland) was used. In ESI positive mode, the mobile phase contained A= water with 0.1% formic acid and B= acetonitrile with 0.1% formic acid; and in ESI negative mode, the mobile phase contained A=0.5 mM ammonium fluoride in water and B= acetonitrile. The gradient was 1%B for 1.5 min and was linearly increased to 99% in 11.5 min and kept for 3.5 min. Then it was reduced to 1% in 0.1 min and a 3.4 min of re-equilibration period was employed. The gradients were at a flow rate of 0.3mL/min, and the column temperatures were kept constant at 25℃. A 2 μL aliquot of each sample was injected.

The ESI source conditions were set as follows: Ion Source Gas1 (Gas1) as 60, Ion Source Gas2 (Gas2) as 60, curtain gas (CUR) as 30, source temperature: 600℃, IonSpray Voltage Floating (ISVF) ± 5500 V. In MS only acquisition, the instrument was set to acquire over the m/z range 60-1000 Da, and the accumulation time for TOF MS scan was set at 0.20 s/spectra. In auto MS/MS acquisition, the instrument was set to acquire over the m/z range 25-1000 Da, and the accumulation time for product ion

scan was set at 0.05 s/spectra. The product ion scan is acquired using information dependent acquisition (IDA) with high sensitivity mode selected. The parameters were set as follows: the collision energy (CE) was fixed at 35 V with ± 15 eV; declustering potential (DP), 60 V (+) and −60 V (−); exclude isotopes within 4 Da, candidate ions to monitor per cycle: 10.

1.2.4 Data processing

The raw MS data (wiff.scan files) were converted to MzXML files using ProteoWizard MSConvert before importing into freely available XCMS software. For peak picking, the following parameters were used: centWave m/z = 25 ppm, peakwidth = c (10, 60), prefilter = c (10, 100). For peak grouping, bw = 5, mzwid = 0.025, minfrac = 0.5 were used. CAMERA (Collection of Algorithms of MEtabolite pRofile Annotation) was sued for annotation of isotopes and adducts. In the extracted ion features, only the variables having more than 50% of the nonzero measurement values in at least one group were kept. Compound identification of metabolites was performed by comparing of accuracy m/z value (<25 ppm), and MS/MS spectra with an in-house database established with available authentic standards.

2. Results

2.1 Weight changes after mice oral exposure to $LaNiO_3$

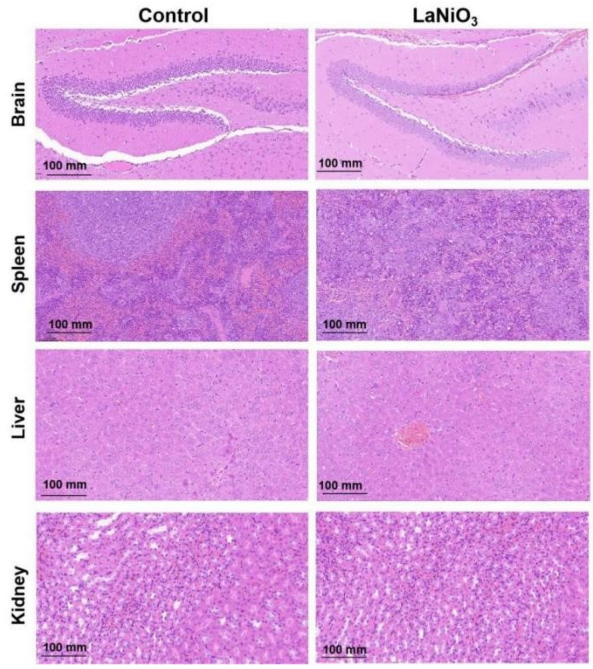

Figure S1 H&E staining to detect the pathology of Brain (100X), Kidney (100X), Liver(100X) and Spleen(100X) in the Control, $LaNiO_3$ and Control group. (*n*=4)

2.2 Perturbance to gut microbiota 7 days after single dose oral exposure to $LaNiO_3$

Table S1 Relative abundance of typical gut bacterial in the measurements for control, $LaNiO_3$ exposed mice of feces. Note: Relative abundance of >0.1% is significant.

Family level			
NAME	Relative abundance Control	Relative abundance LaNiO3	Fold change VS Control
f__Anaeroplasmataceae	2.66E-05	0.001788396	67.23293233
f__Lachnospiraceae	0.017957705	0.451224389	25.12706323
f__Clostridiaceae 1	0.000587525	0.006013135	10.23468789
f__Desulfovibrionaceae	0.000412248	0.004074359	9.88327172
f__Clostridiales vadinBB60 group	0.00092043	0.008671636	9.421287876
f__Helicobacteraceae	0.007889374	0.042914365	5.439514593
f__Prevotellaceae	0.017341755	0.051580083	2.974328896
f__Ruminococcaceae	0.010257294	0.029637967	2.889452813
f__Pseudomonadaceae	0.001644254	0.00266452	1.620503888
f__Bacteroidaceae	0.01901227	0.028089603	1.477446039

Genes level				
NAME		Relative abundance Control	Relative abundance LaNiO3	Fold change VS Control
f__Anaeroplasmataceae	g__Anaeroplasma	2.6556687518629638e-05	0.0017883955206866892	67.34256746
f__Lachnospiraceae	g__Lachnospiraceae FCS020 group	3.404739236024192e-05	0.007035510332084607	206.6387422
f__Lachnospiraceae	g__ASF356	1.1043963522822312e-05	0.0019461089237347857	176.2147185
f__Lachnospiraceae	g__Lachnospiraceae UCG-001	0.00013503993559338802	0.020977593450058275	155.3436275
f__Lachnospiraceae	g__Roseburia	0.00021915855862593607	0.01614232681483483	73.65592709
f__Lachnospiraceae	g__Lachnospiraceae NK4A136 group	0.005878710060444314	0.17851445578543151	30.366263
f__Clostridiaceae	g__Lachnoclostridium	4.2738695615009834e-06	0.0015940698290083661	372.9804586
f__Clostridiaceae	g__Clostridium sensu stricto 1	0.0001883549832267108	0.0023415026483543226	12.43132838
f__Clostridiaceae	g__Ruminiclostridium	0.00046161996396837564	0.004476963891370483	9.698375809
f__Desulfovibrionaceae	g__Candidatus Arthromitus	0.0003912582345317637	0.0036479780321894174	9.32370928
f__Helicobacteraceae	g__Helicobacter	0.007889373925533663	0.04291436541354042	5.439514696
f__Prevotellaceae	g__Alloprevotella	0.008465887922573181	0.037783650521634723	4.463046389
f__Prevotellaceae	g__Prevotellaceae UCG-001	0.008140318060647649	0.012568805021909615	1.54401891
f__Ruminococcaceae	g__Marvinbryantia	4.520182615377661e-06	0.0041795729479114216	924.6469233
f__Ruminococcaceae	g__Oscillibacter	0.00013361704706512737	0.0033194839255734506	24.84326662
f__Pseudomonadaceae	g__Pseudomonas	0.0016442536496990017	0.0026645200928926294	1.62050429
f__Bacteroidaceae	g__Bacteroides	0.01901227011834583	0.02808960285217347	1.477446022

2.3Metabolome changes 7 days after single dose oral exposure to $LaNiO_3$

Table S2 Identified significantly changed metabolites by MS/MS in feces samples in $LaNiO_3$ treated group.Notes：$p < 0.05$ compared to controls.

Name	Fold change	SuperClass
Daidzein 4'-sulfate	13.48226492	Phenylpropanoids and polyketides
7-hydroxyflavanone	6.352298764	Phenylpropanoids and polyketides
Carprofen	6.796341637	Organoheterocyclic compounds
NCGC00169011-01	5.62111822	Organoheterocyclic compounds
4-morpholinopropanesulfonic acid	4.648986014	Organoheterocyclic compounds
1h-indole-1-pentanoic acid, 3-[2-(2-chlorophenyl)acetyl]-	4.398288356	Organoheterocyclic compounds
2-(n-morpholino)ethanesulfonic acid	3.257701696	Organoheterocyclic compounds
Indolelactic acid	3.012802476	Organoheterocyclic compounds
Methanone, (4-hydroxyphenyl)(1-pentyl-1h-indol-3-yl)-	2.882954167	Organoheterocyclic compounds
Ofloxacin n-oxide	2.683097232	Organoheterocyclic compounds
Thiamine monophosphate	2.442126732	Organoheterocyclic compounds
Thymine	2.360717966	Organoheterocyclic compounds
1h-indole-3-propanoic acid	2.102593629	Organoheterocyclic compounds
2,5-dihydroxy-3,6-diphenyl-1,4-benzoquinone	-3.627054748	Organic oxygen compounds
Glyceric acid	-4.685904715	Organic oxygen compounds
Acamprosate	4.216012299	Organic acids and derivatives
Trh, free acid	4.145445161	Organic acids and derivatives
Trandolaprilat	3.967643683	Organic acids and derivatives
Asp-Trp	3.950588044	Organic acids and derivatives
N-acetyl-l-glutamate	3.687307382	Organic acids and derivatives
Taurine	3.440678225	Organic acids and derivatives
Propionic acid	3.433966883	Organic acids and derivatives
Raltitrexed	3.370680539	Organic acids and derivatives
Dl-a-hydroxybutyric acid	2.783870924	Organic acids and derivatives
Thr-Val-Leu	2.598566499	Organic acids and derivatives
(S)-2-Hydroxyglutarate	1.892141317	Organic acids and derivatives
N-acetyl-l-aspartic acid	1.878981054	Organic acids and derivatives
Proline	-2.056105632	Organic acids and derivatives
1-o-octyl-2-o-(n-methylcarbamoyl)-sn-glyceryl-3-phosphorylcholine	8.6619667	Lipids and lipid-like molecules
Deoxycholic acid	4.092413238	Lipids and lipid-like molecules
Echinocystic acid	3.877879793	Lipids and lipid-like molecules
Cinobufagin	3.728038224	Lipids and lipid-like molecules
2-hydroxy-3-methylbutyric acid	3.6607113	Lipids and lipid-like molecules
Hydroxyisocaproic acid	2.882465399	Lipids and lipid-like molecules
alpha-Tocopherol (Vitamin E)	2.676277822	Lipids and lipid-like molecules
Ricinoleic acid	2.605089326	Lipids and lipid-like molecules
Quillaic acid	2.150789443	Lipids and lipid-like molecules
Linolenic acid	-1.946812916	Lipids and lipid-like molecules
Prostaglandin i2	-2.35106131	Lipids and lipid-like molecules
Valerenic acid	-2.562626565	Lipids and lipid-like molecules
N-stearoyltaurine	-2.7061925	Lipids and lipid-like molecules
Ganoderic acid a	-3.275710098	Lipids and lipid-like molecules
1,2-distearoyl-sn-glycero-3-phospho-l-serine	-3.400454299	Lipids and lipid-like molecules
1-palmitoyl-2-oleoyl-phosphatidylglycerol	-4.296538799	Lipids and lipid-like molecules
Fulvestrant 9-sulfone	-32.33410433	Lipids and lipid-like molecules
Imatinib	6.023888214	Benzenoids
Telmisartan	5.23242943	Benzenoids
(2s,3r,4as,12br)-2,3,4a,8-tetrahydroxy-12b-(5-hydroxy-6-methyloxan-2-yl)oxy-3-methyl-2,4-dihydrobenzo[a]anthracene-1,7,12-trione	3.937573412	Benzenoids
Acetylsalicylic acid	3.463782647	Benzenoids
Probucol	3.336520868	Benzenoids
Rac-2-despiperidyl-2-aminorepaglinide	3.004442897	Benzenoids
Tazarotenic acid	7.179152388	Benzenoids
Specnuezhenide	3.96553517	Benzenoids
Hydroxyphenyllactic acid	3.008342938	Benzenoids
Acetoxystachybotrydial acetate	2.745661734	Benzenoids
Phenyllactic acid	2.328586068	Benzenoids
Dl-lactate	2.019188719	Benzenoids
